# The Management of Suppuration, Complicating Tuberculous Disease of the Bones and Joint

**Published:** 1893-03

**Authors:** V. P. Gibney

**Affiliations:** New York


### Southern Medical Record.

A MONTHLY JOURNAL OF MEDICINE AND SURGERY.

VOL. XXIII. ATLANTA, GA., MARCH, 1893. No. 3.

### Original Articles.

### THE MANAGEMENT OF SUPPURATION COMPLICATING TUBERCULOUS DISEASE OF THE BONES AND JOINTS.*

BY V. P. GIBNEY, M. D., NEW YORK.

The time allotted for a discussion of this subject is so short that I have felt it incumbent upon me to-day to make my remarks as brief as possible in order to give the gentlemen who are to follow all the time they need for their papers.

From frequent analyses of cases treated in the hospital, I do not hesitate to present my views without a statistical backing. The claim is often made by my colleagues in orthopedic surgery that suppuration can be prevented by the *timely* use of apparatus. At times I have been strongly tempted to make this claim myself. The whole question hinges on what we understand by the term *timely*. It will be readily admitted, I think, by medical men, as well as surgeons, that, if a case of turberculous bone lesion in the neighborhood of the joint can be recognized very early, before any deformity arises, adequate protection to the joint will certainly contribute largely to the prevention of suppuration. The value of rest to any inflamed tissue has been established too long for any cavil at this late date. A difference of opinion often exists as to what is and

*Read February 8th, 1893, before the State Medical Society of New York.

what is not absolute rest to a part, one surgeon claiming that the apparatus he employs affords perfect protection, even if *absolute rest* is not maintained, while another claims that perfect protection can not be secured unless *absolute rest* is maintained.

Take, for instance, the appliances used in the treatment of tubercular ostitis of the hip, or what is commonly known as "hip disease." The long splint, with which all are familiar and which forms the chief element in the American method of treating the disease, does not absolutely rest the joint, but permits a small range of motion, however snugly applied. The traction employed by means of this apparatus depends largely upon the surgeon himself. Many who use it do not believe that it is possible to get a sufficient amount of traction to separate the articular surfaces, and claim that it gives only a sufficient amount of traction to prevent trauma. On the other hand, there are some who believe that it is possible to make the traction such that the articular surfaces seldom, if ever, come in contact. From a personal knowledge of the manner in which this splint is employed, my own belief is that the protection is seldom perfect, but that it is sufficient to guard the joint against trauma if the surgeon himself looks after the dressings and makes the parent or nurse acquainted with the principle involved. This means, of course, intelligent co-operation. The Thomas splint does not make any traction on the joint, but is simply a fixation appliance, and must of necessity make a lever of the limb, and to do this it is difficult for one to see how the joint can help being injured more or less by contact of articular surfaces. It is claimed that the employment of axillary crutches is sufficient to guard against this evil, yet when one considers how little time during the twenty-four hours the patient is on the axillary crutches, he can readily understand the nature of some of the objections to the apparatus. The combination of the long splint with the Thomas apparatus theoretically affords absolute rest, as well as protection to the joint, and would seem to be all that is desired, yet the difficulty of adjusting and the confinement of the whole body serve to make such a combination apparatus really of little practical value.

It is nevertheless true that with any of these forms of apparatus mentioned, the large proportion of cases taken in the early stage and before deformity has arisen, do proceed to a cure without suppuration and in many instances with complete restoration eventually of the functions of the joint. On the other hand, it is just as true that there are cases taken in the very early stage, with the most approved forms of apparatus and with the most skillful men in charge of the same, that do go on to suppuration in spite of all we may do. We are in the habit of speaking of such cases as severe from the beginning. We speak of the lesion as a large one, involving three or four centres in the end of the bones and rapidly encroaching, one upon the other. Thus much for the prevention of suppuration.

Our knowledge of the nature of tuberculous suppuration in a bone enables us to fix pretty definitely upon the locality, but the location of an abscess is not always a guide to the seat of disease. In small abscesses, attended with very little constitutional disturbance, one can safely treat expectantly, can treat the joint itself by protective apparatus and look upon the pus collection as of minor importance. In the pre-antiseptic day, surgeons as a rule, the world over, were content to leave bone abscesses alone, unless they became acute or very extensive. The surgical rule to *let out pus wherever found* did not apply to cold abscesses, and it was only occasionally that a bold surgeon advocated immediate interference. Since the advent of antisepsis, it has been the custom to incise more freely, and there are many surgeons to-day who believe it good practice to operate whenever a pus collection is found. Yet there are a very respectable number whose experience has been large, who still hesitate to operate unless constitutional symptoms develop.

It may be safely said of the orthopedic surgeon that *early interference is exceptional.* It is claimed by some of these gentlemen that adequate protection to the joint limits the suppurative process and does away with the necessity for incision. A rather extended personal experience has prejudiced me somewhat in favor of the aspiration of small abscesses, without the injection of any fluids or agents. I am satisfied that the percentage of cure is 50 per cent. The introduction of iodoform in the various emulsions has not encouraged me in recommending this procedure.

My plan in aspirating is about as follows: Use a good-sized sterilized needle, go fearlessly and quickly into the sac, evacuate completely if possible, if not, incompletely, apply over the skin a basket strapping of rubber adhesive plaster, the straps drawn tightly, a roller bandage over this, keep the patient in bed for twenty-four hours with an ice bag over the parts, then let him go about with the apparatus properly adjusted. At the end of a fortnight repeat the operation with same after-treatment. From three to six aspirations usually suffice to effect a cure. Where a cure is not effected in this way, I resort to a free incision, expose as much of the sac as possible to view, dissect away or curette with a flush gouge, dry the parts thoroughly with antiseptic gauze, pack lightly with iodoform, dermatol or boracic acid gauze, and over all this a full dressing. Remove the packing in about forty-eight hours, bring the edges of the wound together throughout the greater part of the extent, leaving an opening a half inch in length into which a drainage tube or a gauze tent is inserted, and redress every three or four days. My experience in attempting complete removal of the sac and closure for primary union has not been sufficiently encouraging to lead me to speak favorably of this method. In many instances where I have secured primary union and where the immediate result has been brilliant, the sac has refilled at a later period, and I have been compelled to open the wound anew and do a bone operation, either gouging thoroughly or excising. Where the abscess is in close proximity to the lesion, it is best, in my judgment, to do a bone operation at once; aim to remove the diseased portion and pack the cavity as in an abscess remote from the bone lesion. Roughly speaking, I should say that at least 50 per cent. of abscesses opened in the manner above described, wherein no attempt has been made to treat the bone itself by operative measures, 50 per cent. of these cases, I say, come to the bone operation generally as a life-saving measure. After all, I am not eager to interfere with these small abscesses, and prefer to keep them under observation with protective apparatus to the limb for a long time before interference, and in a fair proportion of cases, the sac grows smaller or becomes more dependent and opens spontaneously with excellent result. So that I am in the habit of recommending to my class at the Polyclinic non-interference, unless the surgeon is fully equipped for details of antiseptic technique.

In the large and multilocular abscess that I meet for the first time, my plan is to incise at once, reduce the cavity if possible to a sinus or sinuses, establish good drainage, and at a later date attack the bone. If, however, the patient's condition is unfavorable, I do not hesitate to attack the bone as soon as I open the abscess. These remarks apply more specially to tubercular disease of the hip joint, and naturally lead up to the question of excision. Notwithstanding the abundance of literature, both statistical and clinical, on this subject, we are not as yet in possession of sufficient knowledge to enable us to determine when to excise and when not to excise. The final results of this operation, so far as I have been able to understand them, do not encourage me to advocate excision in all cases where extensive abscesses are present. In hospital work I have learned to rely upon the condition of the patient, upon his behavior under expectant treatment for a while, upon the condition of the urine, that is, the persistence of a low specific gravity, and upon the probable extent of the disease. The cases most suitable, in my judgment, are those wherein the abscess appears in the gluteal region, is limited to this area, and where one can readily get down upon the bone and secure the best drainage. Such cases, as a rule, present on section a limited area of disease, and whether one attempts complete eradication or partial, results are usually good. The class of cases that are most obstinate and that cause me to hesitate a long time, are those where the abscess appears in the groin, in Scarpa's space, or on the inner side of the thigh. The pus sacs usually encroach extensively upon the femoral vessels, and, as a rule, involve not only the head and neck, but a portion of the shaft. It is next to impossible to remove all the offending material and very difficult to get healing from the bottom by reason of the number of pockets that have formed in the neighborhood of the joint. In a few instances, I have adopted the plan recommended by my friend, Dr. Poore, of New York, namely, curetting the shaft of the femur and making a counter-opening above the knee. My results have not been as good as he has obtained, and hence the measure has not met with my cordial approval. In a few instances, I have curetted the bone without making the counter-opening, have kept the shaft well packed with iodoformized gauze, subsequently with drainage tubes, and a small proportion of such cases have done well, and I feel encouraged to continue this procedure.

The class of cases that are suitable for partial arthrectomy and gouging of bone is very small. In a number of instances I have been compelled to follow with a complete excision weeks or months after the partial operation, and my experience, therefore, coincides with that of surgeons who have written upon this subject.

From a paper recently presented before the Philadelphia County Medical Society on "The Suppurative Stage of Hip Disease," I make the following abstract as supplemental to the remarks already made:

"I succeeded in getting fairly accurate notes of ninety cases, and noted the age of the patient, the date of the appearance of the disease, the date of the appearance of the abscess, the disposition of the abscess, and the result. For present purposes, it is only necessary to make use of the results of treatment, irrespective of the age of the child or the appearance of the abscess. Of this number, then, there were eighteen that opened spontaneously, either at the time of admission or after aspiration. Ten of these, or 55 per cent., did well. That is, the patient suffered no inconvenience, a sinus followed and finally closed. Three, or about 17 per cent., did well also, but a sinus remained. In five there was a failure. That is, the discharge grew very abundant, and it was necessary to resort to other procedures.

"Forty-three were aspirated from one to ten times, and of this number there were four, about 9 per cent., that proceeded to resolution, and thirty-nine failed, or 91 per cent.

"Forty-nine were incised and treated as above described, with twenty-two good results, about 45 per cent., that is, closure of the wound and ultimately of the sinuses. Nine did fairly well, which means the constitutional disturbance was very slight, the wound closed, and only a sinus remained, and eighteen failed, about 37 per cent. This leaves, then, thirty-one or about 63 per cent., that may be set down as successful.

"There were thirty-one cases that were either excised during the past year or are still under observation. Of this number, eighteen, or 58 per cent., did well, that is to say, the wounds have healed and the limbs are in good position and useful for walking, with very little shortening. In nine the result has not yet been reached, but the prognosis is good, and this would give us a possible twenty-seven, which is about 87 per cent. of cases of cure. I do not happen, while writing this paper, to remember the percentage given by other operators, and hence can not be accused of an effort to magnify the good results obtained. Four of the thirty-one cases failed, that is, the patients either died or are in a fair way to die, and this leaves, then, a mortality of nearly 13 per cent. It is also fair to state that this mortality is obtained in cases where the operation was done more frequently as a last resort and as a life-saving measure. I have not yet come to believe that excision should be done early, although I confess that as my experience increases, my convictions are growing stronger in favor of early excision. In a few cases where the operation has been resorted to at an early date the results have been uniformly good, and in some instances brilliant.

"Analyzing the ninety cases still further, I find that in only three instances where a spontaneous opening of the abscess occurred, was it necessary either to incise or excise. In one, incision was employed, or rather, enlargement of the opening with thorough curetting. In two, excision was performed with good results in both cases. In four instances, where aspiration was resorted to, three opened spontaneously at a subsequent date, and generally in the cicatrix from the needle. Three of these were attended with good results ; one was a failure. In sixteen aspiration cases, incision was finally resorted to, with eight good results, six questionable, and two failures. In eight where both aspiration and incision failed, excision was done, with six good results and two failures ; and in eight other cases, where aspiration alone was employed, excision was done, with four good results and four fairly good results. There were five cases where the abscess had previously been incised that were finally excised, with one good result, three fairly good, and one failure. Taking the whole number of cases where aspiration failed, viz.: thirty-nine, thirty-six were subjected to further observation and treatment, with twenty-one good results, ten fairly good results and five failures.

TABLE OF OPERATIONS.

| | | | |
|---|---|---|---|
| Abscesses opening spontaneously... | 18, | with 13 good results | —about 72 per cent. |
| Abscesses aspirated.............. | 43, | with 4 good results | —about 9 per cent. |
| Abscesses incisied................ | 49, | with 31 good results | —about 63 per cent. |
| Cases excised. .................... | 31, | with 27 good results | —about 87 per cent. |

"It would seem, then, from a study of the analysis here presented, that nothing is lost by aspiration, although the percentage of good results is small. It is also apparent that a spontaneous opening is not so undesirable as many are led to believe. If aspiration fails and one decides to incise, rather than excise, we have to console us a percentage of sixty-three wherein a good result was obtained. This means a joint where there is every reason to believe that the disease has undergone resolution and with the limb in good position and sufficiently well ankylosed to make it exceedingly useful for locomotion. All surgeons, I take it, prefer a result of this kind to the average result of excision, for, in the latter operation, one can never tell how much bone must be removed and whether the joint will be substantial or unsubstantial. To obtain the best result, even after excision, I, in common with those who practice orthopedic surgery, believe that the splint treatment should form an important supplement and should be continued for many months thereafter. That which leads up to operation is the dread of exhaustion after prolonged suppuration or an amyloid degeneration of liver and kidneys. The claim that is often made by surgeons that excision shortens the disease, is susceptible of demonstration, for many cases are admitted to the hospital where excision has been performed by a general surgeon long after the operation has been done. These cases come in for the relief of deformity, or for the cure of old sinuses, or for apparatus that will enable them to walk with more ease."

In the management of suppurative disease about the knee joint, I can speak quite confidently of what is known as the expectant plan. That is this: Correct deformity by gradual extension, either in bed with the weight and pulley or with extension apparatus, aspirate when pus is detected, follow this up by incision if the aspiration fails, drain thoroughly, follow this procedure up by gouging out the foci of disease, packing from the bottom, and in every instance, if possible, endeavor to preserve the component parts of the joint. All of my operations upon the knee in children are supplemented by protective apparatus and a long course of such treatment. These remarks apply especially to the disease as it occurs in children. In adults, I favor excision, rather than a partial or complete arthrectomy.

In suppuration involving the bones of the ankle joint in children, it makes little difference whether one incises abscesses or removes foci of disease in the bone, so far as the ultimate result is concerned. I am convinced, from a long study of these cases, that the rule is a recovery with a joint practically perfect. In no instance, I am satisfied, is it ever necessary to amputate the leg for disease of the ankle joint in children. This is not true of a like disease in adults. Where one can be satisfied that bones within reach are alone involved, then a partial operation, with fixation of the joint for many months thereafter, does result in a cure. Yet one can never feel satisfied that all of the bones diseased have been reached, and hence the necessity for more stringent measures.

I have said nothing thus far on the tuberculous disease of the bones of the spinal column, but the same treatment is applicable here as in the bones already mentioned. I do believe that if one treats the back with a splint for a long period of time, that the abscess sacs are easily managed and that in a large percentage of cases a cure will result. By splinting the back, I mean the continuous and uninterrupted employment of an apparatus that will prevent motion and deformity. It is not sufficient to apply a brace or a jacket that can be removed at will by the parents or their friends, for the reason that abuses are bound to occur and disappointment will follow.

In concluding this desultory paper, let me summarize as follows:

(1.) Protect the joint, about which the bone lesion exists, in the early stage and in the later stages, whether the abscess is let alone, aspirated or incised.

(2.) In cases where the suppurative process is confined to a small area, it is good surgery to leave the small abscesses alone if the protective appliance is adequate.

(3.) It is good practice to aspirate where the abscess is in the way of the proper adjustment of apparatus, and by such procedure, one may expect good results in at least 50 per cent.

(4.) The simple incision of an abscess dependent upon bone disease depends for good result upon the extent of the bone lesion.

(5.) Excision of the hip is not a measure to be employed in all cases where extensive suppuration exists, but must depend largely upon the condition of the patient and the location and extent of the abscesses.

(6.) Expectant treatment for the knee and ankle joint in children yields the best results for life and limb.

(7.) Amputation of the ankle in a child is rarely ever justifiable; of the knee, is justifiable only when amyloid disease of liver or kidneys threatens or is present; of a hip, after a thorough excision has failed.

(8.) The long continued employment of a good fitting splint to the back in Pott's disease of the spine, will yield better results than any operative procedures on the bone with which I am familiar.

